# Full resolution reconstruction of whole-mount sections from digitized individual tissue fragments

**DOI:** 10.1038/s41598-024-52007-5

**Published:** 2024-01-17

**Authors:** Daan Schouten, Jeroen van der Laak, Bram van Ginneken, Geert Litjens

**Affiliations:** 1 Department of Pathology, Radboud University Medical Centre, Nijmegen, The Netherlands; 2 Department of Radiology, Radboud University Medical Centre, Nijmegen, The Netherlands

**Keywords:** Computational biology and bioinformatics, Medical research

## Abstract

Whole-mount sectioning is a technique in histopathology where a full slice of tissue, such as a transversal cross-section of a prostate specimen, is prepared on a large microscope slide without further sectioning into smaller fragments. Although this technique can offer improved correlation with pre-operative imaging and is paramount for multimodal research, it is not commonly employed due to its technical difficulty, associated cost and cumbersome integration in (digital) pathology workflows. In this work, we present a computational tool named PythoStitcher which reconstructs artificial whole-mount sections from digitized tissue fragments, thereby bringing the benefits of whole-mount sections to pathology labs currently unable to employ this technique. Our proposed algorithm consists of a multi-step approach where it (i) automatically determines how fragments need to be reassembled, (ii) iteratively optimizes the stitch using a genetic algorithm and (iii) efficiently reconstructs the final artificial whole-mount section on full resolution (0.25 µm/pixel). PythoStitcher was validated on a total of 198 cases spanning five datasets with a varying number of tissue fragments originating from different organs from multiple centers. PythoStitcher successfully reconstructed the whole-mount section in 86–100% of cases for a given dataset with a residual registration mismatch of 0.65–2.76 mm on automatically selected landmarks. It is expected that our algorithm can aid pathology labs unable to employ whole-mount sectioning through faster clinical case evaluation and improved radiology-pathology correlation workflows.

## Introduction

**Figure 1:**
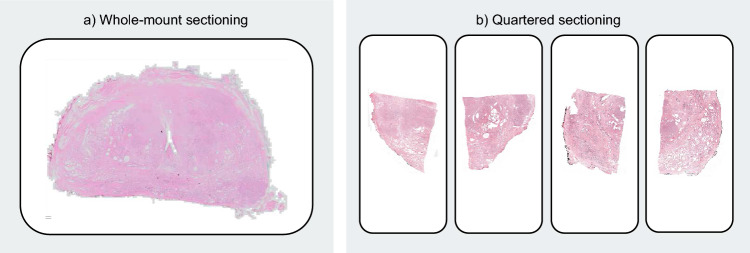
Comparison of (**a**) whole-mount sectioning and (**b**) quartered sectioning for a transversal cross-section of a prostatectomy specimen.

In the field of histopathology, excised tissue can be processed and assessed either as whole-mount sections (WMS) or in a more fragmented approach (Fig. 1). Although the WMS technique is applicable to various organs^1,2^, it is most prevalent in the histopathological evaluation of prostate cancer^3^. The utilization of WMS offers several advantages, particularly an improved correlation between histopathology and pre-operative imaging^2,4^, a reduction in tissue cutting artifacts, and the preservation of tissue context^5^. Through these advantages, the availability of WMS can facilitate diagnostics by potentially improving the efficiency and accuracy of the pathologists’ work. Furthermore, the availability of WMS is indispensable for research studies (e.g. radiology-pathology correlation studies), as evidenced by the multitude of imaging studies employing WMS as a reference standard^6–12^. However, despite these advantages, adopting whole-mount histopathology encounters various practical limitations. Firstly, WMS require larger microscope glass slides due to the larger tissue area. These larger microscopy glass slides are generally double the width of regular slides and may not always be available in pathology labs since these tend to increase the complexity of archival slide storage^2^. Additionally, even when these larger glass slides are available, they may still be slightly undersized for some larger prostates, necessitating further sectioning on a case-by-case basis. Furthermore, the pathology labs that have switched to digital pathology^13^ encounter the practical limitation that most whole-slide scanners are limited to regularly sized microscopy slides and cannot scan the larger slides resulting from WMS. Lastly, there is some evidence that the preparation of WMS requires more expertise and technical proficiency than quartered sectioning^2,5^, but this is not entirely undisputed^2^. The substantial impact of these practical limitations was corroborated by a working group aimed at establishing consensus on radical prostatectomy sampling, which demonstrated that only 16% of 148 respondents reported using WMS for sectioning of radical prostatectomy specimens^5^.

Hence, given these practical limitations, the advantages of WMS are currently inaccessible to most pathology labs. However, the advent of digital pathology presents a potential remedy. In digital pathology, physical slides are scanned with dedicated scanners to obtain a high resolution (0.25 µm/pixel) digital image of the slide^14^. With the growing adoption of digital pathology^13,14^ and with increasing amounts of tissue fragments being digitized, there is an avenue for researchers to develop algorithms that digitally stitch tissue fragments into an artificial WMS resembling the original cross-section of the specimen. These artificial WMS are expected to provide similar benefits as actual WMS, while being more accessible to labs where routine processing of WMS is infeasible. Indeed, preliminary evidence suggests that artificial WMS can reduce pathologist reading time and interobserver variability^15^. Furthermore, artificial WMS have proven to be an indispensable tool in radiology-pathology correlation studies^4,16–19^.

However, the development of these stitching algorithms is non-trivial given the characteristics of whole-slide images (WSI). Firstly, the image size of a WSI scanned at the highest resolution, usually 0.25–0.50 µm/pixel, can easily surpass 100,000 pixels in both width and height, which is prohibitively large for even trivial image processing tasks such as rotating the image by several degrees. Secondly, in contrast to image stitching in panoramic photos^20^, WSI do not exhibit any degree of overlap, rendering stitching based on overlapping landmarks infeasible. Lastly, processing and handling tissue fragments often induces some degree of morphological distortion, thereby limiting the use of solutions based on direct curve matching^21^. Despite these challenges, several tissue stitching algorithms have been proposed^15,22,23^, of which *HistoStitcher*^22^ and *AutoStitcher*^23^ are of particular significance given their specific development for prostate WMS and their public availability.

HistoStitcher can be considered the first algorithm developed for reconstructing WMS from individual tissue fragments and was specifically developed for prostate WMS where four prostate quadrants must be stitched to emulate an artificial WMS. To reconstruct the artificial WMS, HistoStitcher required a pathologist to annotate corresponding landmarks on adjacent fragments which were subsequently used to compute the coordinate transform to stitch the fragments. Although this approach proved more efficient than generic software tools such as Adobe Photoshop, manual annotation of landmarks was cumbersome and introduced interobserver variability. The need for a more automated tool eventually led to the development of AutoStitcher, which employed a genetic algorithm with a domain-inspired cost function to stitch adjacent fragments, thereby eliminating the need for manual landmark annotation. Although AutoStitcher solved most of HistoStitcher’s limitations, it still had several drawbacks. Firstly, although the stitching was performed automatically, AutoStitcher still required manual labeling of the fragment’s location (i.e. upper-left or lower-right quadrant) in the original cross-section. This hinders the use of AutoStitcher for larger datasets where pathology reports with the sectioning reference may not be available and manual labeling is too time-consuming. Furthermore, AutoStitcher could not reconstruct the artificial WMS in full resolution due to memory constraints, and the common use-case of stitching two halves instead of four quadrants was not supported. Lastly, AutoStitcher was developed in proprietary software (MATLAB^24^), not maintained through a version-controlled repository, and incompatible with contemporary software versions. With the introduction of PythoStitcher, we aim to address all of the aforementioned limitations and provide a new baseline for reconstructing artificial WMS. In this paper, we describe the different components of PythoStitcher and provide validation on multiple datasets demonstrating its effectiveness. The source code of PythoStitcher is publicly available on GitHub (https://github.com/computationalpathologygroup/pythostitcher) and we provide a containerized version of our algorithm for long-term access and usability. In addition, we release several example cases on Zenodo (https://zenodo.org/record/8093632) for reproducibility of our results.

## Results

### Automatic fragment configuration

The main results of the automatic fragment configuration are summarized in Table 1. We consider a reconstruction to be successful once the correct configuration is present in any of the suggested reconstructions. For all test set cases with four fragments we observed a median of four and maximum of six suggested reconstructions. We report the accuracy of the fragment configuration in twofold, once for the fully automatic approach and once when taking small manual adjustments into account. For the fully automatic approach, PythoStitcher obtains the correct reconstruction in 68.2–82.7% across the different test sets. The failure cases of this approach can generally be attributed to one of four categories: a wrong prediction of the fragment classifier, a fragment was horizontally flipped during slide preparation, failure to accurately detect the stitch edge or failure to identify the correct solution as a feasible solution. The first two of these categories represent the majority of all failure cases and can be easily solved with a slight manual correction, namely manually specifying the location of the stitch edge or horizontally flipping the culprit fragment before running PythoStitcher, respectively. Taking these manual corrections for initial failure cases into account, this notably improves the configuration accuracy to 86.4–100% across all test sets.

**Table 1:** Evaluation of the automatic fragment configuration for the different datasets.

Dataset	Accuracy without manual correction	Manual corrections	Accuracy with manual correction	Fragment configuration time (min)	Stitching time (min)
Development set	83.3%	4 (7%)	90.0%	2.41 (0.46)	1.80 (0.52)
Internal test set 4	82.7%	9 (9%)	90.8%	2.47 (0.83)	1.93 (0.86)
Internal test set 2	81.8%	6 (18%)	100%	0.03 (0.01)	1.93 (0.52)
External test set	68.2%	4 (18%)	86.4%	2.07 (0.51)	1.64 (0.56)
Pancreas test set	n.a.*	n.a.*	100%	n.a.*	3.32 (0.98)
Artificial test set	73.3%	8 (27%)	100%	1.63 (0.24)	1.52 (0.44)

A manual correction is defined as either manually specifying the location of the stitch edges or horizontally flipping one of the fragments before running PythoStitcher. The values for fragment configuration time and stitching time are displayed as median (IQR).

*For the pancreas test set the location of the stitch edge was always manually specified resulting in only one feasible solution to stitch these fragments and omission of the fragment configuration phase.

**Figure 2:**
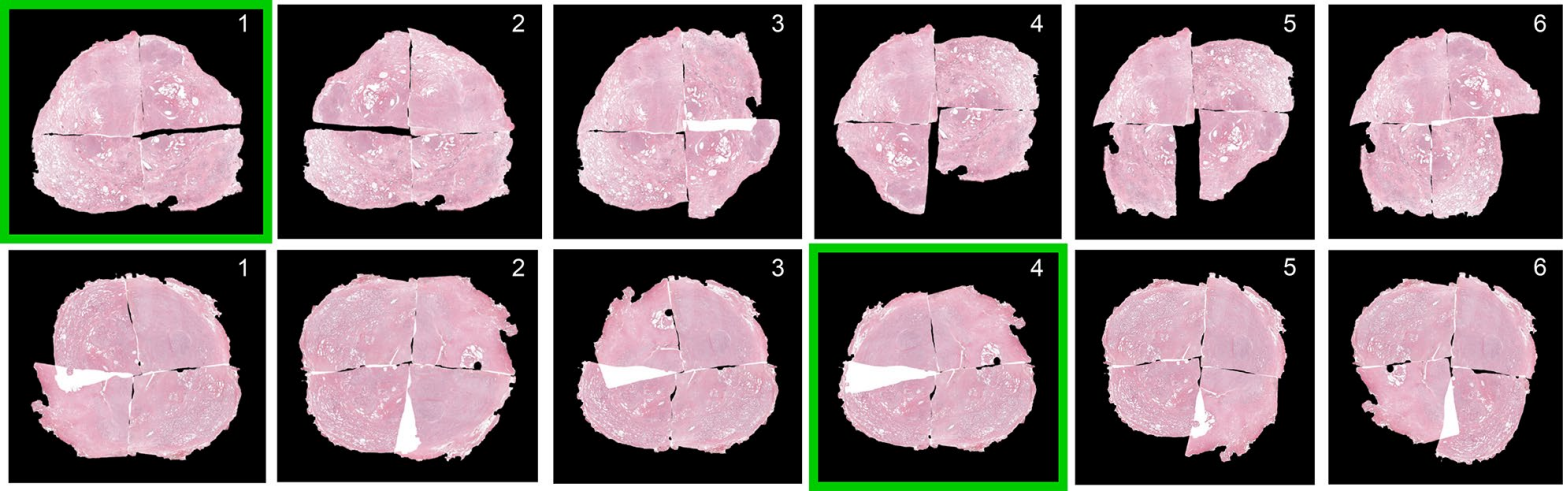
Example of the solution ranking from two prostatectomy cases with four fragments with the correct solution annotated in green. All solutions were scored and ranked on the mean registration error between stitch edges before any optimization with the genetic algorithm. Overlapping areas between adjacent fragments are indicated in white. The top row shows an example where the correct solution had the lowest registration error and thus the highest rank. The bottom row shows an example where the correct solution was ranked fourth due to a substantial registration error originating from significant overlap between the upper left and lower left quadrant.

**Figure 3:**
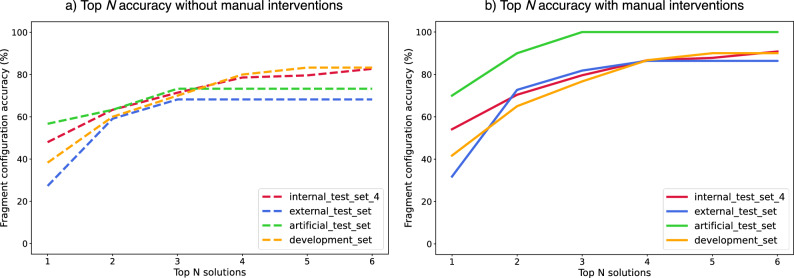
Fragment configuration accuracy when only taking the top N ranked solutions into account, shown for both the case (**a**) without manual correction and (**b**) with manual correction. This figure aims to demonstrate the trade-off between increased accuracy and the approximate linear increase in computational overhead with every additionally included solution.

Lastly, we show that PythoStitcher efficiently narrows down the initial solution space of 64 configurations by not suggesting more than six feasible fragment configurations per case. Based on the available computational resources, the end user can then choose to generate the full-resolution end result of either a selection or all of these solutions. Since these feasible solutions are ranked in likelihood, we can compute the final configuration accuracy by propagating the top *N* feasible solutions for full-resolution reconstruction. An example ranking of feasible solutions for two representative prostatectomy cases with four fragments is visualized in Fig. 2. These examples highlight the difficulty of fully automatically selecting the correct solution, as swapping two adjacent fragments may lead to a seemingly correct solution with a very similar mean squared error, such as in solution one and two for the case in the upper row. Nevertheless, Fig. 3 demonstrates that the solution likelihood ranking by PythoStitcher is able to identify most correct solutions and can be used to notably reduce computational overhead at the expense of a slightly lower configuration accuracy.

### Multi-resolution approach

**Figure 4:**
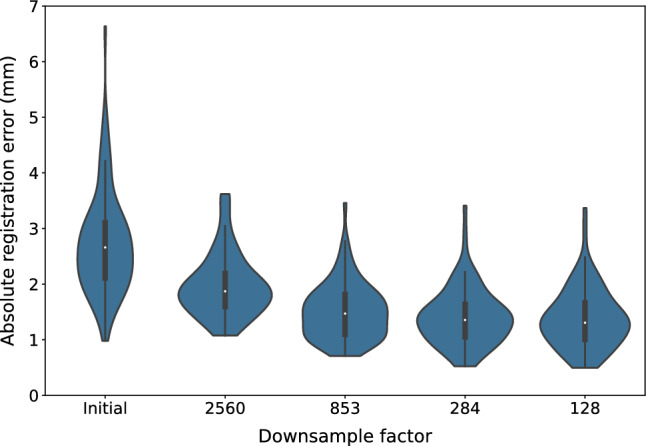
Violin plot of the absolute registration error for internal test set 4 as measured on a rough initial initialization and four different resolution levels. The labels on the x-axis indicate by which factor the original WSI was downsampled where "initial" refers to the rough first stitch initialization without optimization by the genetic algorithm at a downsampling factor of 2560.

The added value of our multi-resolution approach is quantitatively visualized in Fig. 4. This figure displays how the mean residual registration error for internal test set 4 steadily decreases after optimization at different resolution levels. Although the genetic algorithm was run to convergence at each resolution level, it appears that a single resolution level is insufficient to fully optimize the stitch between fragments. This is also in concordance with our visual inspection of the results, which show that the lowest resolution is used to quickly close the gap between adjacent stitch edges and the higher resolutions are used to finetune the exact fit between fragments.

### Stitching accuracy

**Figure 5:**
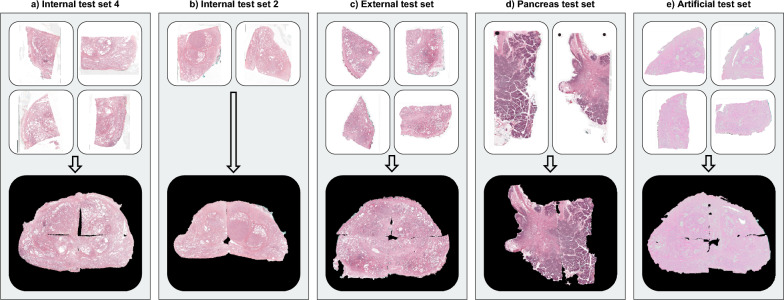
Representative example case for each test set showing the individual fragments and the final stitched result.

The quantitative stitching accuracy was computed for all cases where PythoStitcher identified the correct solution and is summarized in Table 2. The stitching accuracy on the artificial test set is hereby of particular importance since this registration error was computed using ground truth landmarks and serves as a fully objective evaluation. To put the absolute error into context, Table 2 also displays the relative registration error which was computed by dividing the absolute error by the mean WMS image dimensions. Figure 5 demonstrates a representative reconstruction for all test data sets where the absolute registration error of these examples is in close proximity to the median for that particular dataset.

**Table 2:** Quantitative stitching results for the different datasets.

Dataset	WMS size (mm)	Absolute registration error (mm)	Relative registration error (mm)
Development set	36 × 49 (27 × 47, 39 × 62)	1.11 (0.77)	2.46% (1.54)
Internal test set 4	39 × 45 (30 × 40, 50 × 56)	1.31 (0.66)	3.07% (1.47)
Internal test set 2	28 × 38 (25 × 28, 36 × 44)	1.23 (0.91)	3.76% (2.70)
External test set	33 × 45 (28 × 32, 38 × 54)	1.60 (0.71)	4.16% (1.00)
Pancreas test set	34 × 48 (29 × 36, 44 × 51)	2.76 (2.57)	6.94% (7.12)
Artificial test set	29 × 42 (20 × 36, 36 × 55)	0.83 (0.83)	2.16% (1.75)

WMS size is reported as median (range) and registration errors are reported as median (IQR). Note that only the artificial dataset possessed ground truth landmarks for registration error computation, for the other datasets landmarks were automatically detected and the subsequent registration error should be considered an approximation.

### Computational efficiency

When assessing computational overhead for the correctly stitched cases, we demonstrate that both the fragment configuration and the optimization with the genetic algorithm are achieved in a few minutes (Table 1). It should be noted that the stitching time refers to the stitching time per suggested solution and the total stitching time is therefore approximately a multitude of this number depending on the number of suggested solutions that were propagated from the automatic fragment configuration. For example, the total computational overhead for a case with three suggested solutions amounts to the fragment configuration time plus three times the stitching time. In addition, there is some computational overhead in writing the full- resolution stitched WMS to disk, but this greatly depends on external factors such as network speed and computer usage. Empirically, we found that saving a 5GB stitched WMS at 0.25 µm/pixel resolution takes approximately one hour and saving a 35MB stitched WMS at 4.0 µm/pixel resolution takes a few minutes.

## Discussion

In this work, we have presented PythoStitcher, a stitching tool to reconstruct artificial WMS from tissue fragments to aid clinical assessment of prostatectomies and to facilitate radiology-pathology correlation workflows. We demonstrated that PythoStitcher can accurately reconstruct the original WMS in the vast majority of cases over a range of diverse datasets. Furthermore, our computationally efficient algorithm design and algorithm containerization promotes reproducibility of our results on regular clinical workstations. Hence, it is expected that PythoStitcher will be able to bring the benefits of WMS to pathology labs which are currently unable to process these WMS.

When comparing our results with the state-of-the-art method AutoStitcher, we demonstrate similar stitching accuracy in the range of a 2–3% relative registration error based on automatically detected fiducial points and a potentially faster stitching speed for cases with four quadrants. In addition to the previously mentioned additional features of PythoStitcher (i.e. full resolution reconstruction), we also note that PythoStitcher can be used on a substantially wider range of prostatectomy cases. Whereas *Penzias *et al*.* stated that 43/256 (17%) cases in the AutoStitcher cohort had to be excluded due to their hemispherical instead of quartered sectioning^23^, we demonstrate that PythoStitcher can also stitch these hemispherical cases with similar accuracy to quartered cases. Furthermore, *Penzias *et al*.* stated that 100/256 (39%) cases in their cohort had to be excluded due to missing tissue or extra-prostatic tissue. In our combined cohort of the development set and internal test set 4, we excluded 9/167 (5%) cases due to tissue artefacts such as missing tissue or extraprostatic tissue. Although *Penzias *et al*.* do not elaborate on their relatively high exclusion rate, we speculate that this is partially due to their envisioned method for automatic fragment rotation. While they approach the automatic fragment rotation through a rule-based method, our approach is mainly powered by a convolutional neural network which is likely more robust in edge cases of oddly shaped fragments, resulting in our comparatively low exclusion rate. Lastly, a contrasting feature of PythoStitcher is that its cost function is solely tissue shape inspired and does not require any further image information during optimization, rendering it essentially a staining-agnostic solution.

One of the primary limitations of this work is that PythoStitcher cannot automatically reconstruct WMS with an arbitrary number of fragments, as this would greatly increase the complexity of the automatic fragment configuration due to the exponential increase in potential matches between fragments. However, for scenarios with more than four fragments where the stitching task can be divided into multiple batches of two or four fragments, PythoStitcher can be employed iteratively to reconstruct the WMS. For example, in a case with six evenly sized fragments one could run PythoStitcher separately for a group of four fragments and a group of two fragments, after which these intermediate results can be stitched to obtain the final result. Nevertheless, future research could explore the potential for extending PythoStitcher to an arbitrary number of fragments, similar to the problem of shredded natural image reconstruction^28^. This would likely also improve generalizability for different tissue types as this would have to satisfy the requirement of stitching fragments from varying shapes and sizes. Another limitation is that the fully automatic approach in the fragment configuration may fail to find the correct configuration in some cases, requiring a manual correction from the end-user. For example, for several cases we encountered that one of the fragments was accidentally flipped during preparation of the microscope slide, leading to one mirrored image which prohibited a correct reconstruction. If PythoStitcher is employed in a routine diagnostic setting, preventing flips during slide preparation would be an important step that could further improve PythoStitcher’s performance. Alternatively, given that PythoStitcher is already equipped with an automatic fragment configuration, this component could be extended to consider additional configurations with one or multiple flipped fragments at the expense of increased computational overhead. Lastly, we note that PythoStitcher is essentially staining-agnostic due to its design to perform the stitching purely based on tissue masks rather than image characteristics. Although this greatly enhances PythoStitcher’s generalizability, it also emphasizes the importance of having access to high quality tissue masks in a clinical workflow. Hence, before implementing PythoStitcher in the digital pathology workflow, it is imperative that its integration is coupled with a high quality tissue segmentation algorithm to ensure accurate stitching results.

In conclusion, we have presented an efficient algorithm for reconstructing WMS from individual digitized tissue fragments. It is expected that usage of these artificial WMS will assist clinicians through a shorter case evaluation time and stimulate multimodal research through improved radiology-pathology correlation workflows.

## Methods

**Figure 6:**
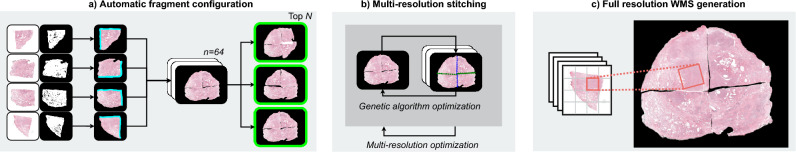
Schematic overview of the entire reconstruction pipeline for an example prostatectomy case with four fragments. Compartment (**a**) represents the automatic fragment configuration where the stitch edges of each fragment are extracted, all possible configurations (64 in the case of 4 fragments) are computed and evaluated to find the top *N* best solutions. Compartment (**b**) displays how the top *N* solutions are finetuned with a genetic algorithm and emphasizes the multi-resolution iterative aspect. Compartment (**c**) demonstrates the tile-based approach where the final full-resolution reconstruction is computed tile by tile.

The general working mechanism of PythoStitcher is schematically visualized in Fig. 6 and can be divided in three distinct components, namely (i) the automatic fragment configuration, (ii) multi-resolution stitching and (iii) generating the full-resolution WMS. As algorithm input, PythoStitcher requires either two or four tissue digitized tissue fragments and their corresponding tissue segmentation mask. These tissue segmentation masks can be obtained through image processing operations such as Otsu thresholding or advanced deep learning based methods^25^. The remainder of this section will elaborate on the three algorithm components and describe the datasets used for validating PythoStitcher.

### Automatic fragment configuration

The automatic fragment configuration entails the process of finding the correct location and orientation of each of the input fragments. Since tissue specimens can be rotated and/or flipped during microscope slide preparation and pathology reports may not always be available to infer the original location of a fragment in the macro block, reassembling multiple fragments into an artificial WMS is a non-trivial task. PythoStitcher aims to automate this reassembly task, thereby eliminating the need for manual fragment labeling or manual stitching instructions. In essence, this reassembly task consists of identifying the exact border along which these fragments need to be stitched, also known as the stitch edge of a given fragment, and identifying adjacent fragments. Compartment (a) in Fig. 6 shows these stitch edges highlighted in cyan and how these stitch edges are subsequently used to explore different potential configurations. This automatic fragment configuration in PythoStitcher is mainly powered by a convolutional neural network (EfficientNet-B0^26^) which classifies where the stitch edges in a fragment are located (i.e. the upper/lower border and right/left border). PythoStitcher then uses this information to compute the exact coordinates of the stitch edges of all fragments. Next, the reconstruction is essentially treated as a jigsaw puzzle and an exhaustive list of all possible fragment configurations is explored by aligning the different fragments along their stitch edges on a heavily downsampled version of the fragment. In the case of four prostate quadrants as input images where each fragment has two stitching edges, this results in 64 (4^3^) ways to reconstruct the whole-mount as indicated by the stack of images in compartment a) of Fig. 6. This exhaustive list is then filtered by a set of two constraints where only potentially feasible reconstructions are retained. First, all fragments must connect in a loop where each stitch edge of a fragment is connected to one stitch edge of an adjacent fragment, thereby ensuring an ellipsoid shape of the reconstruction which emulates the natural shape of the prostate. Second, an empirical threshold allowing a maximum of 20% overlap between adjacent fragments filters reconstructions which adhere to the first constraint but are highly unlikely due to shape mismatches between adjacent fragments. All feasible reconstructions are then ranked based on the mean Euclidean distance between stitch edges and, depending on the available computational resources, the top *N* highest ranking or all of the feasible reconstructions are propagated to the next component of multi-resolution stitching. Hence, the output of this component consists of multiple suggested reconstructions to increase the chance of retrieving the correct solution at the expense of a few incorrect or false positive reconstructions.

Note that for the scenario of two fragments, there is only one single option to align the fragments once the stitch edge is known. Hence, this automatic fragment configuration step is bypassed in the case of two fragments. Additionally, it should be noted that the user retains the freedom to optionally enforce a fragment configuration for the fragments (i.e. left and right or specific quadrants), which is helpful in cases with oddly shaped fragments where automatic stitch edge detection may be difficult.

### Multi-resolution stitching

For each of the top *N* configurations from the automatic fragment configuration, PythoStitcher aims to optimize the stitch through a multi-resolution stitching approach with increasingly higher resolution versions of the fragments. The stitching process is generally difficult to optimize since an improved stitch between two fragments may deteriorate the stitch quality between the other adjacent fragments, invoking a long recursive process of optimization. PythoStitcher aims to tackle this problem by employing a genetic algorithm (using the PyGAD^27^ library) to iteratively optimize the stitch between multiple fragments at once. This is accomplished by posing the problem as a task where the optimal rotation and translation for all but one fragment need to be obtained, resulting in (*n* − 1) ∗ 3 degrees of freedom where *n* is the total number of fragments. The cost function *C*_*pair*_ for two adjacent fragments *f*_1_ and *f*_2_ is defined as the mean Euclidean distance between the coordinates of *k* evenly sampled landmarks *p* along the stitch edge of both fragments:

1 $$\begin{array}{c}{C}_{pair}\left({f}_{1},{f}_{2}\right)=\frac{1}{k}{\sum }_{i=1}^{k}||{p}_{{f}_{1},i}-{p}_{{f}_{2},i}{||}_{L2}\end{array}$$

The total cost *C*_*total*_ for a given solution *S* in the genetic algorithm with *j* adjacent pairs of fragments *f*_*a*_ and *f*_*b*_ can then be defined as:

2 $$\begin{array}{c}{C}_{total}\left(S\right)=\frac{1}{j}{\sum }_{i=1}^{j}{C}_{pair}\left({f}_{a},{f}_{b}\right)\end{array}$$

For each iteration in the genetic algorithm, PythoStitcher evaluates the fitness of 40 solutions and picks the two best solutions to propagate into the next generation. The next iteration in the genetic algorithm will then consist of the two aptly named parent solutions and 38 minor variations of these parent solutions, where a variation refers to a slightly different rotation and/or translation for any of the fragments. An upper bound on the maximum allowed difference in rotation and/or translation was set to stimulate a gradual improvement rather than extreme variations in the solution space. This iterative process of evaluating all solutions per generation is then repeated until either the predefined maximum number of iterations is completed or the best solution has failed to improve for 50 consecutive generations as defined by the cost function. This optimization with the genetic algorithm is then performed over four different resolution levels of the fragment starting with the lowest resolution. Compared to the full resolution WSI, these different resolution levels refer to a downsampling factor of 2560, 853, 284 and 128 where the latter equals a resolution of 32 µm/pixel. After completion of the optimization for a given resolution level, the best solution is propagated and the genetic algorithm is repeated for the higher resolution level. With this higher resolution there is now room for additional optimization with the genetic algorithm since the exact location of the landmarks which guide the stitching process can be more accurately computed. Effectively, the efficient lower resolutions aim to achieve the bulk of the optimization and the more computationally costly higher resolutions serve to finetune this previously obtained solution.

### Full resolution reconstruction

Since the image size of the individual fragments is generally in the order of gigabytes, reconstructing an artificial WMS from multiple of these large images requires a dedicated computational pipeline. To enable WMS reconstruction at a resolution as high as 0.25 µm/pixel, PythoStitcher leverages a tile-based approach using the PyVips^28^ library where the final reconstruction is computed in a tile-based manner. This approach entails that the WSI is split into a large number of small tiles (e.g. images of size 64 × 64) after which the optimal rotation and translation for that fragment obtained from the multi-resolution stitching component are applied individually to all these tiles. In essence, this splits up the computationally infeasible task to rotate and translate the full WSI into multiple smaller transformations that are computationally feasible while obtaining the exact same transformed WSI. With this approach, the required computational overhead only depends on the tile size and not the image size of the WSI. In principle, this allows PythoStitcher to reconstruct the artificial WMS at arbitrarily high resolutions and image sizes without any memory bottlenecks. Hence, this enables PythoStitcher to be employed on regular clinical workstations rather than requiring dedicated compute infrastructure.

**Figure 7:**
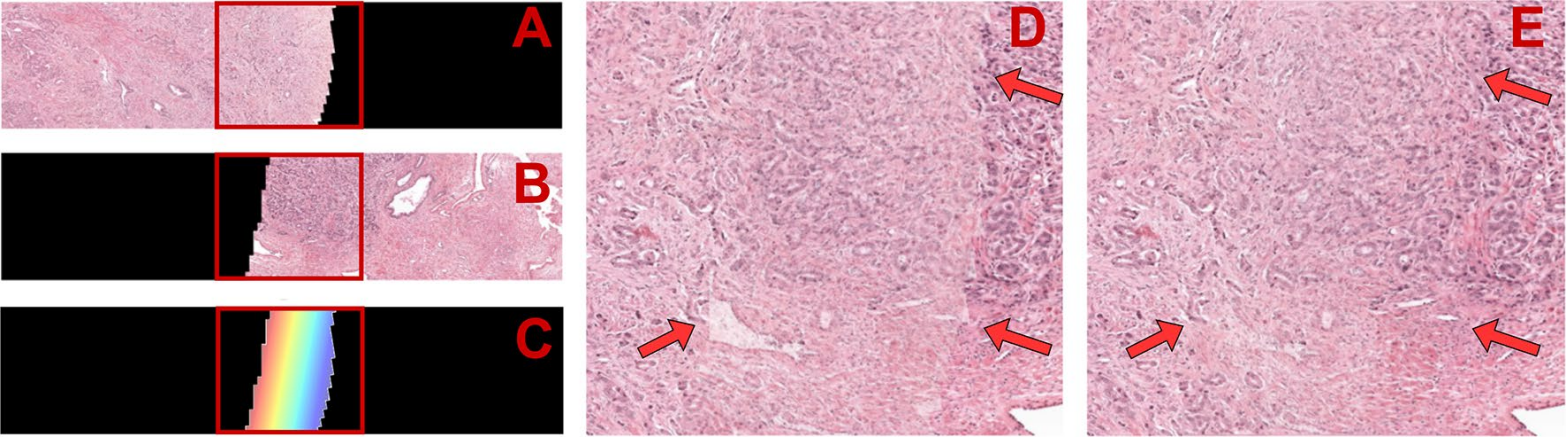
Schematic overview of the proposed blending method. A and B show a small tile of two individual fragments which pose a slight overlap in the final reconstruction. C displays the region of overlap and the gradient that is computed to facilitate the gradient blending as described in Eq. (3). D shows a magnified version of the area marked by the red box in A-C where the final image is computed by averaging the pixel intensities from fragment A and B. The red arrows indicate how this results in clear boundary artefacts at the edge of the overlapping area. E shows our proposed gradient blending method where the boundary artefacts seem visually mitigated.

An additional feature of the full-resolution reconstruction is that PythoStitcher blends overlapping areas of adjacent fragments, leading to a more visually appealing stitch edge between fragments. Although larger areas of overlap are anatomically unfeasible, some minor overlapping areas are to be expected since individual tissue fragments undergo slight deformations during preparation and handling of the microscopy slide. Furthermore, an individual tissue fragment cut from one block may originate from a slightly different depth of the block, in the range of several µm, compared to a tissue fragment cut from the adjacent block, thereby further preventing a perfect stitch. Hence, the aforementioned factors combined with the use of a rigid transformation will seldom lead to a perfect interlock between adjacent fragment boundaries resulting in minor overlapping areas. To handle these overlapping areas, PythoStitcher generates a transition between fragments where the pixel intensity at a given location is a weighted average of the pixel intensity of both fragments at that location. This weighting is computed proportionally to the proximity of the location to both fragments, such that a pixel in the overlapping area closer to fragment A will more closely resemble the appearance of fragment A than fragment B. Mathematically, we define the pixel intensity *I* at location (*x,y*) in the overlap area as:

3 $$\begin{array}{c}I\left(x,y\right)=\alpha \left(x,y\right){I}_{A}\left(x,y\right)+\left(1-\mathrm{\alpha }\left(x,y\right)\right){I}_{B}\left(x,y\right)\end{array}$$

where *α*(*x,y*) represents the proportional weighting applied to the pixel intensity in fragment A *I*_*A*_(*x,y*) and the pixel intensity in fragment B *I*_*B*_(*x,y*). Figure 7 demonstrates a comparison between a non-weighted averaging of pixel intensities versus the employed blending method.

### Experiments

#### Fragment classifier

To train the model to predict the stitch edges of a given fragment, this task was formulated as a multiclass classification problem with four different labels. Based on clinical prostate sectioning we made the assumption that each fragment is roughly shaped as a quarter ellipse, implying that two stitch edges are always adjacent and never on opposite sides of the fragment. The four labels can then be regarded as the location of both stitch edges, i.e. left and top or right and bottom. Annotation of the location of the stitch edges in all cases was generally trivial and performed by a researcher in computational pathology. The training data consisted of one randomly chosen quadrant per patient of 146 patients from Radboud University Medical Center (Nijmegen, the Netherlands) and 133 patients from Rijnstate Hospital (Arnhem, the Netherlands). Each quadrant was downsampled to 16 µm/pixel, padded with whitespace to achieve a square shape and resized to 512 by 512 pixels. Data augmentation primarily consisted of random horizontal and vertical flips and rotations by 90 degree increments. The model was an EfficientNet-B0^26^ pretrained on ImageNet and was finetuned using fivefold cross-validation with the Adam optimizer and categorical crossentropy as loss function. The learning rate was set to 1e-4 for the first 15 epochs after which it was decreased to 1e-5 for 30 more epochs or until the validation accuracy failed to improve for 5 consecutive epochs. After training the model was externally validated on 52 patients from Erasmus University Medical Center (Rotterdam, the Netherlands). The patients on which the model was trained were excluded from the final stitching evaluation of PythoStitcher for an unbiased evaluation of the automatic fragment configuration component.

#### Stitching accuracy

For the retrospective evaluation of PythoStitcher we perform our validation on various datasets to demonstrate robustness in different scenarios. The primary characteristics of these datasets are summarized in Table 3. The WSI from the artificial test set were digitized with an Olympus VS100 scanner at 0.32 µm/pixel resolution. All other WSI were digitized with an 3DHISTECH P1000 scanner at 0.25 µm/pixel resolution. Note that a separate development dataset consisting of 60 prostatectomy cases with four fragments from the Radboud University Medical Center (Nijmegen, the Netherlands) was used to tune all (hyper)parameters in PythoStitcher. Hence, the cases from the test sets in Table 3 can be considered unseen data on which PythoStitcher was not optimized. We used the automatic fragment configuration for all datasets, except for the pancreas dataset. Since pancreas surgery is more variable, pancreas sectioning is not as systematic and structured as prostate sectioning. This resulted in the pancreas fragments exhibiting a larger variety of shapes, making automatic stitch edge detection infeasible. Hence, the pancreas fragments were manually labelled according to their original location in the specimen, either the left or right side.

**Table 3:** Main characteristics of the datasets used for the development and evaluation of PythoStitcher.

Dataset	Anatomy	Institute	Number of patients	Number of fragments per patient
Development set	Prostate	Radboud University Medical Center (Nijmegen, The Netherlands)	60	4
Internal test set 4	Prostate	Radboud University Medical Center (Nijmegen, The Netherlands)	98	4
Internal test set 2	Prostate	Radboud University Medical Center (Nijmegen, The Netherlands)	33	2
External test set	Prostate	Rijnstate Hospital (Arnhem, The Netherlands)	22	4
Pancreas test set	Pancreas	Radboud University Medical Center (Nijmegen, The Netherlands)	15	2
Artificial test set	Prostate	Radboud University Medical Center (Nijmegen, The Netherlands)	30	4

Internal test set 4 and 2 refer to data with four and two pieces of tissue.

**Figure 8:**
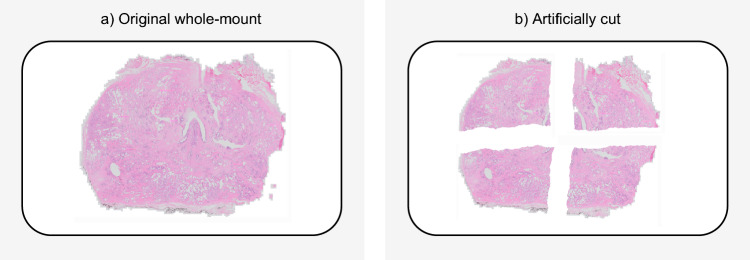
Example of a whole-mount (left) which was artificially cut into four different fragments. Note how the edges along which the fragments were cut are not fully straight and mimic the slightly curved edges of the fragments obtained from the quartered sectioning as displayed in Fig. 1.

Since the cases from the upper five datasets in Table 3 all originate from sectioned specimens, there is no ground truth WMS and the quantitative evaluation is an approximation rather than an exact computation. Hence, we also introduce an artificial test set where we use 30 actual prostatectomy WMS and digitally cut these into four different fragments per WMS such that it closely resembles the prostatectomies with quartered sectioning (Fig. 8). Care was taken to closely mimic the fragment shape from fragments in the internal and external test set to ensure that reconstructing the WMS in this artificial test set was of similar difficulty. As the cutting was done digitally, we can use the exact location of landmarks to validate PythoStitcher’s stitching accuracy in a truly objective manner. For the remaining datasets, we approximate the stitching accuracy by automatically selecting ten landmarks along pairs of adjacent fragments and computing the absolute registration error in mm as previously described in Eqs. (1) and (2). These landmarks were selected by choosing ten evenly spaced points on the stitch edge for a given fragment. The absolute registration error was chosen to provide an intuitive metric on the stitching quality and to facilitate indirect comparison with previous methods^23^. Furthermore, in contrast to more general image registration tasks, we do not have any overlapping structures in adjacent fragments, rendering the more classical structure-based (i.e. Dice coefficient) and intensity-based (i.e. mutual information) registration metrics infeasible. PythoStitcher’s performance on all datasets was assessed on a Linux machine with an Intel Xeon e5-2630 CPU, an NVIDIA GeForce RTX 2080 Ti graphics card, and 16 GB RAM.

### Ethics statement

During their cancer treatment, patients were informed that left-over tissue material can be used for research, and at that time, they had no objections to such use. The Institutional Review Board (IRB) of the Radboud University Medical Center therefore waived the need for informed consent for this study (2022–15,878) and approved the use of anonymized data for training and validating the algorithm. This also includes the data from the Rijnstate Hospital and Erasmus Medical Center since this data fell under the same application that was evaluated by the IRB. This research was performed in accordance with the Declaration of Helsinki.

## Data Availability

For each of the datasets used in this paper one representative case can be obtained through https://zenodo.org/record/8093632. The full datasets are available from the corresponding author upon reasonable request.

## Code availability

The full algorithm code is publicly available at https://github.com/computationalpathologygroup/pythostitcher.
